# Seeing through the veil: blue-light filtering and peak contrast sensitivity in pseudophakic patients

**DOI:** 10.1007/s00417-026-07171-x

**Published:** 2026-03-10

**Authors:** Jacob B. Harth, Lisa M. Renzi-Hammond, Spencer F. Smith, Billy R. Hammond

**Affiliations:** 1 Institute of Gerontology, College of Public Health, The University of Georgia, 102 Spear Road, Athens, GA 30602 USA; 2 Vision Sciences Laboratory, Behavioral and Brain Sciences Program, Department of Psychology, The University of Georgia, 120 Baldwin Street, Athens, GA 30602 USA

**Keywords:** blue-haze, visual range, intraocular lens, blue light filters

## Abstract

**Purpose:**

Atmospheric short-wave scattered light (blue haze) limits visual range by reducing the contrast of distant objects. While theoretical models and prior research suggest that filtering short wavelengths can improve visual range, significant individual differences persist in tolerance to veiling luminance, even among individuals with similar ocular health and acuity. This study examined whether a commercially available blue-light filtering (BLF) lens design could enhance visual range under simulated blue haze conditions in an older pseudophakic population.

**Methods:**

Forty older pseudophakic adults (*M* = 71.15 ± 5.82 years) were tested using a randomized, controlled, cross-over design. Visual range was assessed via a custom two-channel optical system presenting a sinusoidal grating target veiled by adjustable blue haze (Correlated Color Temperature = 9424 K). Participants viewed the target through a clear control lens and a test BLF lens in randomized order. Visual range thresholds, defined as the maximum blue haze energy tolerated before target detection failed, were determined using the psychophysical method of limits.

**Results:**

Participants tolerated significantly greater (*U* = 1035.00; *p* = 0.024) average blue haze energy with the BLF lens (3.49 log relative energy [LRE]) than with the clear control lens (3.43 LRE). On average, the BLF lens allowed participants to withstand approximately 19% more veiling luminance before target obscuration.

**Conclusions:**

Consistent with theoretical predictions and prior findings in younger adults, BLF lens designs significantly enhanced visual range in older pseudophakic participants under simulated blue haze conditions. These findings suggest that BLF lenses may offer functional visual benefits in older adults, especially in populations with intraocular lens implants lacking native blue-light filtration. Future research should extend these results using field studies in outdoor environments.

## Key messages

*****What is known***:**

- Short-wavelength light scattering ("blue haze") reduces visual range by lowering the contrast of distant objects.
- Filtering short wavelengths can enhance visual range, but prior studies have only tested younger, healthy adults.

*****What is new***:**

- In older pseudophakic adults, blue-light filtering (BLF) lenses significantly improved visual range under simulated blue haze conditions.
- Participants tolerated ~19% more veiling luminance with BLF lenses compared to clear control lenses.

## Introduction

Atmospheric short-wave (blue) scattering has a significant influence on the ability of individuals to see distant objects. As light traverses the atmosphere, shorter wavelengths within the visible spectrum, particularly blue and violet light, are scattered more efficiently by air molecules and fine particulate matter due to their inverse fourth-power dependence on wavelength (Rayleigh’s equation). In practice, this has often been quantified using the Koschmieder Eq [1]., which relates object contrast to atmospheric extinction (scattering and absorption):

1 $$\:C={C}_{0}\cdot\:{e}^{-\beta\:x}$$

where:

*C* = contrast of the object at distance x

*C*_*0*_ = intrinsic contrast of the object (without haze)

*β* = atmospheric attenuation coefficient (depends on scattering and absorption)

*x* = distance to the object

Preferential scattering causes distant objects to appear progressively less distinct, with diminished sharpness and contrast as viewing distance increases. Additionally, the scattered short-wavelength light contributes to a characteristic bluish tint, often described as the depth cue aerial perspective. The cumulative result is a perceptual limitation on the maximum observable range, whereby distant objects are obscured by a diffuse atmospheric veil of scattered light. This limit (*V*, where contrast drops below threshold, typically around 0.05) has also been quantified.

2 $$\:V=\frac{3}{\beta\:}$$

where:

*V* = limit where contrast drops below threshold.

*β* = atmospheric attenuation coefficient (depends on scattering and absorption).

**Fig. 1:**
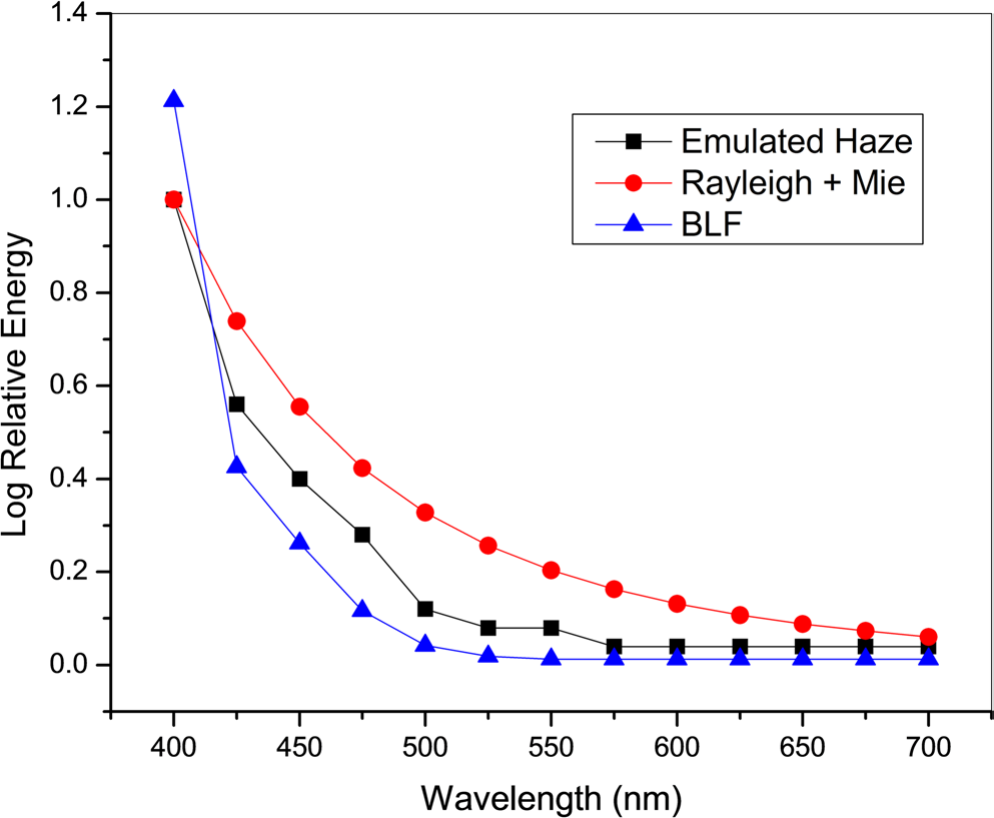
A comparison between the emulated blue haze (squares, black line) and a combination Rayleigh + Mie Scatter function (circles, red line) with a scattering coefficient of ƛ^−5^ [11]. The optical density of the test blue light filter is shown with the blue line (triangles). The “haze” produced by the optical system was well matched to the percentage of light scattered via (mostly) a Rayleigh function. Note that the spectral absorbance of the blue-light filter (BLF) we used (expressed here as optical density) fits both of these curves fairly well (hence, selective filtering represents a straightforward mechanism for how BLFs of this sort could improve visual function under these spectral conditions)

This equation integrates the effects of both Rayleigh (molecular) and Mie (aerosol/particle) scattering (a typical haze spectrum is shown in Fig. 1). For a hazy day, a *β* value might be about 0.1 km [2]. Hence, when trying to view a distant object, based on Equ. 2, the absolute distance would translate to about 30 km (about 19 miles).

These equations offer good approximations. It is also known, however, that large individual differences exist in how far one can see even when most variables (age, visual acuity, etc.) are equivalent [3]. Extensive modeling [4] and empirical data [5, 6] suggest that one primary factor driving these differences is filtering by intraocular pigments that absorb short-wavelength light, such as macular pigment (MP). MP optical density (MPOD) can range near 0 to over 1.0 at their peak absorbance, which impacts short-wavelength light available at the retina to be transduced. For example, Hammond et al. [7] reported on a participant whose peak MPOD was 1.63, which means that at 460 nm, only 2.3% of the light was transmitted to the underlying cones.

Improved visual range certainly has adaptive value, and the ability to accumulate MP may be an adaptation that supports seeing through atmospheric haze. However, not all modern humans have high MPOD, and they could therefore benefit from other means of achieving similar improvements in visual range. One strategy would be adding short-wave “blue-light” filtering (BLF) to contact lenses, intraocular lenses (IOL), or spectacles. Many products aimed at athletes already add filtering to goggles/spectacles for this purpose [8]. Such approaches, however, while faster than dietary change to increase MPOD, do leave some questions unanswered. For example, what is the effect of screening the entire retina as opposed to just cones in the macula, like MP? MP maximally absorbs at 460 nm, but that is relatively far into the visible spectrum; what is the effect of confining filtering to 400–440 nm? Previous studies [5, 6] have also focused on only young healthy participants. Do the same benefits also generalize to older adults? In this study, we address these questions by testing 40 older adults using a clear control lens and a BLF lens (a common spectral filtering profile was used; see [9]).

## Methods

### Participants

Participants were recruited from two ophthalmic practices in the Athens, Clarke-County area following medical records review by the study optometrist. Of the 59 participants who expressed interest in participation, 10 participants were lost to follow-up after the study visit was scheduled, and 9 participants did not meet inclusion criteria. Forty participants (*M* = 71.15 ± 5.82 years; range = 60–86 years; 57.5% female; 82.5% White) met inclusion criteria and attended the study visit. Descriptive characteristics for the sample are displayed in Table 1.

Inclusion criteria required that all participants were 18 years of age or older, had written and verbal fluency in English, could sit for 20 min without breaks, were not pregnant or lactating, had uncorrected Snellen visual acuity of 20/40 or better in one or both eyes, could see the test stimuli without wearing additional vision correction, were in good ocular health deemed by the study optometrist and recruiting practices, were implanted with a clear IOL (either model SA60WF or CC60WF) in the test eye, and participated within a window of two-months to two-years after cataract surgery was complete. Participants who failed to meet the inclusion criteria were excluded from participation. Individuals with either model SA60WF or CC60WF IOLs were eligible, but only individuals with CC60WF lenses participated.

All study materials and procedures were reviewed and approved by the Sterling Institutional Review Board (Atlanta GA, USA; Study ID 11865), with local context provided by the University of Georgia Human Research Protection Program. All participants provided written and verbal informed consent prior to their participation in the study. The tenets of the Declaration of Helsinki were adhered to throughout the completion of the study.

**Table 1:** Descriptive Characteristics of the Study Sample

	*n*	*%*	
Gender			
Male	17	42.5	
Female	23	57.5	
Race			
White	33	82.5	
Black or African American	6	15.0	
Asian	1	2.5	
Implanted Eye			
O.D.	8	20.0	
O.S.	2	5.0	
O.U.	30	75.0	
Ocular Dominance			
O.D.	25	62.5	
O.S.	15	37.5	
Iris Hue			
Grey	8	20.0	
Blue	13	32.5	
Green	3	7.5	
Hazel	7	17.5	
Brown	9	22.5	
Iris Lightness			
Light	22	55.0	
Medium	12	30.0	
Dark	6	15.0	
	**Mean**	**SD**	**Range**
Age (years)	71.15	5.82	60–86
Ishihara Color Test*	16.49	2.27	3–17

*Out of a maximum score of 17

### Materials and apparatus

A custom-built two-channel optical system was adapted from the device previously described in Hammond et al. [10] and was used to measure visual range. The target consisted of a 3.0 cycles per degree sinusoidal contrast grating (0.2 cycles per millimeter grating 86 cm from the eye of the observer) illuminated by 520 nm light produced by two 520 nm lasers that each traveled through their own integrating sphere to ensure homogenous illumination. The output of these two spheres, one of which passed through a glass sinusoidal grating and an optical chopper set to a rate of 1 Hz, was combined using a beam splitter. A photodetector was used to ensure output of each sphere (grating [0.20 cd/m2] and background [3.00 cd/m2]) remained constant. The luminance of this target was relatively dim as one would expect a distant target to be when viewing it outdoors. The second channel in this system, which produced the “blue” veiling haze, used a 150 W xenon lamp as the light source (ThorLabs; Model SLS401) which was passed through an adjustable neutral density wedge. A “blue-sky” filter was used to yield a veiling stimulus with spectral characteristics (Correlated Color Temperature = 9424 K) that closely matched a combination of Rayleigh and Mie scattering (scattering coefficient of ƛ^−5^) to approximate environmental blue-haze [11].

These two channels were combined using a glass beam splitter which allowed for the experimenter to control the level of blue haze that veiled the sinusoidal contrast grating (which was kept constant). Optical baffling was used to prevent light interference between or within the channels. The participant used a forehead and chin rest to stabilize their head while viewing the sinusoidal grating target (~ 86 cm away from eye of the observer) through the beam splitter (~ 58 cm from the eye of the observer). Prior to each test session, spatial alignment of each channel was checked and luminance for all stimuli and veils was recorded (UDT Instruments; Model S370 Optometer).

Two extraocular lenses were tested (the transmission spectra are provided in Hammond et al. 2025). One lens was visibly clear and served as the control lens. The other lens (Fig. 1) absorbed violet-blue with a spectrum that resembles the commonly implanted Clareon/AcrySof Natural IOL absorbance profile. This study utilized a single-masked, controlled, cross-over design with lens order randomized.

### Procedure

The intensity of the veiling blue haze was initially set to a low level, so the grating was clearly visible. Participants were then positioned in the chin and forehead rest and asked to view the sinusoidal grating target through the low-level emulated veiling haze while wearing the first randomly selected lens (test or BLF). Lenses were mounted in trial lens frames that were adjusted to comfortably fit their faces. The sinusoidal grating target contrast and luminance remained constant while the researcher slowly increased the intensity of the veiling haze using the neutral density wedge (psychophysical method of limits). The amount of energy transmitted was recorded and logged (base 10) and expressed as log relative energy (LRE) values (a higher number translates to a participant being able to see through more haze before losing sight of the grating target). Participants indicated when they could no longer resolve the grating. A minimum of three trials were completed before the participant was fitted with the second randomly selected lens.

### Statistical analyses

Statistical analyses were conducted using SPSS (Version 29; IBM). Frequencies and percentages were calculated for categorical variables while means and standard deviations were calculated for continuous variables. Levene’s test was used to determine whether variances were homogeneous, and the Shapiro-Wilk test was used to determine whether distributions were normal. Distributions for both the clear (*W* = 0.917, *p* = 0.006) and BLF lens (*W* = 0.882, *p* < 0.001) were skewed. Consequently, the Wilcoxon Signed Ranks test was completed to determine whether LRE for the veiling haze was significantly different between the test and control lens. A *p* < 0.05 was used as the criterion for statistical significance.

## Results

**Fig. 2:**
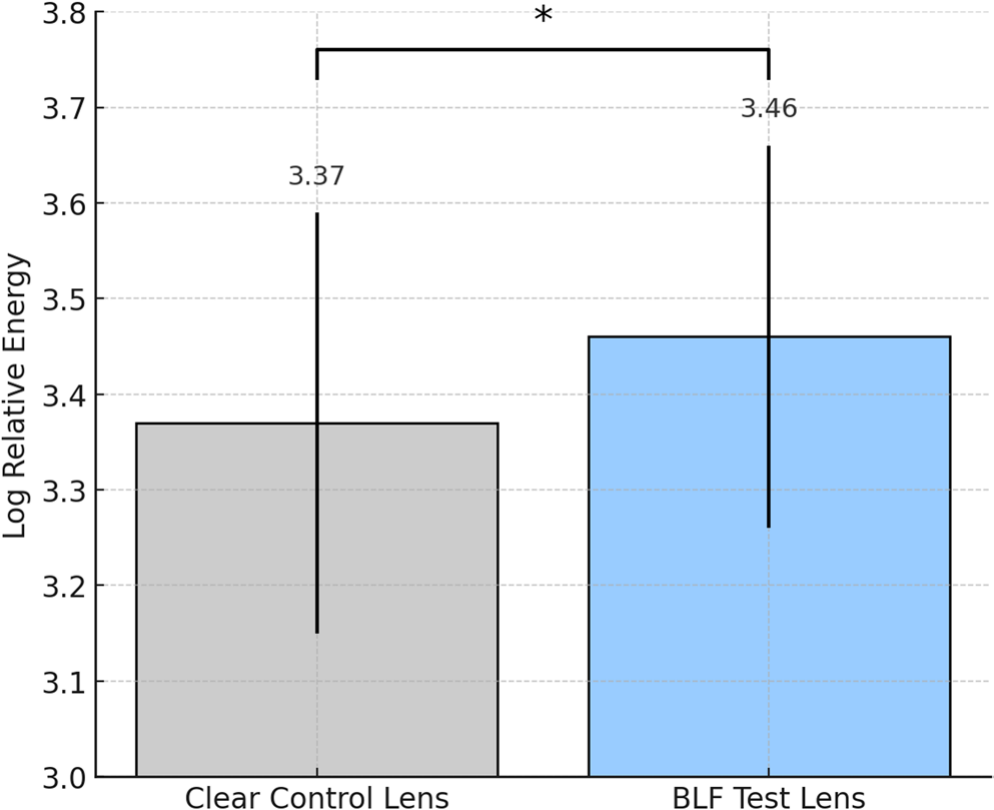
The energy needed to veil a grating target for the clear control lens and the blue light filter. These values in log relative energy (LRE) are expressed as the amount of energy (logged, base 10) needed (19% when expressed as a linear ratio) to obscure a grating in the participant’s line of sight. A statistically significant difference in LRE values between the blue light filter (BLF) and clear control lens conditions (*z* = 5.399, *n* = 40, *p* < 0.001, *r* = 0.854) was found

As shown in Fig. 2, the mean LRE of the blue veil required to occlude the sinusoidal grating target was 3.46 ± 0.20 (median = 3.49) for the BLF lens and 3.37 ± 0.22 (median = 3.42) for the clear control lens. This represented an average increase of approximately 0.09 log units when participants viewed the target through the BLF lens compared to the clear control lens. When converted to linear values, this difference translates to approximately a 19% increase in haze energy tolerance before the grating could no longer be resolved. These differences were significant (*z* = 5.399, *n* = 40, *p* < 0.001, *r* = 0.854). Figure 2 displays the distribution of individual LRE values for both lens conditions, highlighting the consistent upward shift in performance when viewing through the BLF lens.

## Discussion

The present study examined the influence of a BLF lens with a similar profile to a common, commercially available IOL on visual range under emulated blue haze conditions in pseudophakic older adults. Consistent with theoretical predictions based on Rayleigh and Mie scattering principles [4] and prior empirical research [5, 6], our findings demonstrate that BLF lenses can improve the ability to detect a grating target under blue veiling luminance conditions. Specifically, participants wearing the BLF lens were able to tolerate approximately 19% more veiling haze energy before target detection thresholds were reached, as compared to a clear control lens.

These results extend our prior findings in younger populations, which focused on MP as an internal BLF [4, 6]. The finding that extraocular BLF are also useful in older participants (*M* @ 71 years) is notable and perhaps even counterintuitive. Older individuals have significant increases in intraocular scatter [12], less sensitivity to short-wave light [13], and high lens density at the shortest visible wavelengths [14]; all factors which could potentially make additional short-wave light filtering problematic. Another interpretation, however, is that all these factors may simply translate as a noisier system at the shortest wavelengths, or at least a system more susceptible to perturbation. By improving signal-to-noise ratios, BLF may restore visual range under these intense, high short-wave lighting conditions.

Stressful lighting conditions are common in the modern world. New vehicle headlights often cause visual disability and discomfort for drivers at night, and glasses made for nighttime driving frequently contain BLFs which can reduce glare indices and positive dysphotopsias [9]. Many sports professionals and outdoor workers already utilize BLF spectacle lenses to enhance visual performance [8]. Our study suggests that these benefits likely extend to older adults and could be particularly valuable in populations needing IOL implants, where native short-wavelength filtration may be altered depending on the IOL’s spectral profile. All participants in this study had CC60WF IOLs, which do not possess the same blue-light filtration properties as other IOL models [15]. Thus, external BLF lenses may offer an important adjunctive benefit in this population.

One limitation of the present study is the use of a laboratory-based emulated haze environment. It is certainly possible to emulate the spectral form of haze, but capturing the complexity of natural lighting and atmospheric conditions, which change dynamically over time and across seasons, is not possible. Future studies should attempt to validate these findings in real-world field settings (although the distances and dynamic atmospheric conditions involved may make reliable measurements in the environment difficult).

In conclusion, our results are consistent with past studies showing that BLF can significantly enhance visual range under blue-haze conditions, even in older adults. These findings contribute to a growing body of literature emphasizing the importance of strategically modifying incident light, not only on overall neurological health [16, 17] but also optimizing visual function, especially in older adults. Filtering blue light may reduce dysphotopsias, improve behavioral light scatter, reduce glare disability and discomfort [18], and, based on these data, increase visual range under blue haze conditions.

## Data Availability

The data that support the findings of this study are available from the corresponding author upon reasonable request.
